# Mitochondrial DNA and Distribution Modelling Evidenced the Lost Genetic Diversity and Wild-Residence of Star Tortoise, *Geochelone elegans* (Testudines: Testudinidae) in India

**DOI:** 10.3390/ani13010150

**Published:** 2022-12-30

**Authors:** Shantanu Kundu, Tanoy Mukherjee, Ah Ran Kim, Soo-Rin Lee, Abhishek Mukherjee, Won-Kyo Jung, Hyun-Woo Kim

**Affiliations:** 1 Department of Marine Biology, Pukyong National University, Busan 48513, Republic of Korea; 2 Agricultural and Ecological Research Unit, Indian Statistical Institute, Kolkata 700108, India; 3 Research Center for Marine Integrated Bionics Technology, Pukyong National University, Busan 48513, Republic of Korea; 4 Agricultural and Ecological Research Unit, Indian Statistical Institute, Giridih 815301, India

**Keywords:** testudines, wildlife trafficking, conservation genetics, suitable habitat, translocation, conservation

## Abstract

**Simple Summary:**

Genetic diversity and habitat suitability of the star tortoise, *Geochelone elegans* is poorly understood throughout its range in South Asian countries. The mitochondrial gene sequence and distribution modeling analyses demonstrated lower intraspecific genetic diversity and more highly fragmented habitats in India than in Sri Lanka. The present study recommends the intensive genetic screening of wild and trade/captive individuals before translocation, effective enforcement to prohibit wildlife trafficking, and habitat restoration urgently demanded to conserve this highly-threatened species in the wild.

**Abstract:**

The Indian star tortoise (*Geochelone elegans*) is a massively traded animal in South Asia. To mitigate this risk, the conservation agencies recommended guidelines to safeguard this charismatic species in nature. We adopted mitochondrial DNA-based investigation and performed species distribution modeling of *G. elegans* throughout its distribution range in the Indian subcontinent. The genetic analyses revealed weak genetic landscape shape interpolations, low intraspecific distances (0% to 1.5%) with mixed haplotype diversity, and a single molecular operational taxonomic unit (MOTU) in the cytochrome b gene dataset. The star tortoise, *G. elegans*, and its sister species *Geochelone platynota* showed a monophyletic clustering in the Bayesian (BA) phylogeny. We also attempt to understand the habitat suitability and quality of *G. elegans* in its distribution range. Our results suggest that, out of the extant area, only 56,495 km^2^ (9.90%) is suitable for this species, with regions of highest suitability in Sri Lanka. Comparative habitat quality estimation suggests the patch shape complexity and habitat fragmentation are greater in the western and southern ranges of India, which have been greatly influenced by an increased level of urbanization and agriculture practices. We have also provided a retrospect on the potential threat to *G. elegans* related to the wildlife trade on the regional and international spectrum. Our results detected multiple trading hubs and junctions overlying within the suitable ranges which need special attention in the vicinity. The present study calls for a proper conservation strategy to combat the fragmented distribution and explicitly recommends intensive genetic screening of founder individuals or isolated adult colonies, implementing scientific breeding, and subsequent wild release to restore the lost genetic diversity of star tortoises.

## 1. Introduction

The Indian star tortoise, *Geochelone elegans* is a medium-sized reptile species classified under the family Testudinidae (order Testudines). The species was originally described under the genus *Testudo* and later on transferred to *Geochelone* with its closest relative, the Burmese star tortoise (*Geochelone platynota*) [1]. This land tortoise can be easily distinguished by its unique geometric pattern of carapace and plastron with light radiating lines on a dark background and vice versa. The star tortoise prefers to live in the grasslands and scrub forests of arid and semi-arid regions. The species is largely herbivorous; nevertheless, they are also known to scavenge on animal matter and play an important role in ecosystems [2]. In the recent past, *G. elegans* confronts habitat loss, anthropogenic threats, and severe menaces due to illegal hunting, with approximately one hundred thousand individuals traded every year [3,4]. Hence, the species is enlisted to CITES ‘Appendix I’ in 2019 to prohibit all international trade and safeguard them in the wild. The Tortoise and Freshwater Turtle Specialist Group (TFTSG) categorized *G. elegans* under the ‘vulnerable’ category in the IUCN Red List of Threatened Species [5].

Several studies have aimed to comprehend the morphology, ecology, captive care, and breeding of *G. elegans* from different regions [6,7]. More recently, a few molecular studies have been conducted with the intent to illuminate the genetic diversity of *G. elegans* in a restricted manner. The first approach aimed to elucidate the phylogenetic assessment of *G. elegans* (live and trade materials) through nuclear and mitochondrial DNA analyses [8]. Consecutively, three mitochondrial protein-coding genes were analyzed to clarify the genetic diversity of *G. elegans* from Pakistan, India, and Sri Lanka, and a comprehensive systematic screening was recommended across the entire distribution range [9].

In response to illegal wildlife trafficking, successful enforcement often involves immediate confiscation of animals and their holistic management to make them born free [10]. To avoid the risk of losing the genetic makeup and other attributes of confiscated animals, conservation organizations (CITES, IUCN, WWF, etc.) have recommended several guidelines for the management and placement of confiscated or live organisms [11,12,13,14]. The TFTSG member declares that *G. elegans* is endemic to the Indian subcontinent and demarcated its distribution range in three broadly isolated geographic areas viz., north-western India and adjacent south-eastern Pakistan, southern and south-eastern India, and Sri Lanka [5]. However, recognition of the most suitable habitat within the distribution range for the release of confiscated *G. elegans* into the wild for sustainable conservation is still poorly known.

The genetic data has been evidenced as a successful tool in conservation genetics and facilitating the rapid return of trafficked turtles back to the wild [15]. In this milieu, the present study aimed to examine the genetic diversity of *G. elegans* throughout its distribution range. In addition, species distribution modeling is often able to develop substantial knowledge of the habitat and biogeography of many chelonian species under past, current, and future climate conditions [16,17,18]. These spatial data also provide an appropriate means of reintroducing many threatened species after successful breeding or confiscation and help establish effective conservation management [19,20]. Owing to the guidelines of a multilateral conservation treaty, the present study further aimed to determine the suitable habitat of *G. elegans* through distribution modeling (SDM) to forecast a piece of vital information regarding the prioritized area for developing conservation and management in the Indian subcontinent.

## 2. Materials and Methods

### 2.1. Sampling and Ethics Statement

The biological samples were collected from 13 star tortoises, kept locally as pets, from seven different localities. After communicating with the pet keepers, we came to understand that seven individuals were collected from the inside of the known distribution range (20.30 N 85.76 E, 19.83 N 85.77 E, 17.40 N 78.62 E, and 13.61 N 78.44 E); however, the remaining were collected outside of this range (26.16 N 91.69 E, 26.42 N 88.25 E, and 22.60 N 88.37 E). The species was identified as *G. elegans* based on its unique geometric pattern of carapace with light radiating lines on a dark background and plastron with dark radiating lines on a light background [2]. The biological samples (a drop of blood) were collected from the hind limb of each individual using a sterile needle and preserved in the blood collection cards. All experiments were performed in accordance with the relevant guidelines and regulations of the host institutes and ARRIVE 2.0. (https://arriveguidelines.org) rules [21]. No ethics committee or institutional review board approval was required for this work as no animals were killed or encountered in the wild.

### 2.2. Molecular Experiments

The genomic DNA was extracted using a Qiagen DNeasy Blood & Tissue Kit (QIAGEN Inc., Germantown MD, Hilden, Germany). To compare the genetic diversity from earlier and present distant localities, a partial fragment of the cytochrome b (Cytb) gene was amplified using a published primer pair (mcb398/mcb869) [22]. The PCR was performed in a 25 µL reaction mixture of 1× PCR buffer, 2 mM of MgCl_2_, 10 pmol of each primer, 0.25 mM of dNTPs, 0.25 U of high-fidelity polymerase, and 20 ng of template DNA in a Veriti Thermal Cycler (Applied Biosystems, Waltham, MA, USA) with specific thermal profiles. The amplified DNA was purified using a QIAquick Gel Extraction Kit (QIAGEN) following the standard protocol. The cycle sequencing was performed using a BigDye Terminator v3.1 Cycle Sequencing Kit (Applied Biosystems) with 3.2 pmol of each primer. Subsequently, the PCR products were purified using a BigDye X-terminator kit (Applied Biosystems, Waltham, NJ, USA) and sequenced bi-directionally by a 3730 Genetic Analyzer (Applied Biosystems, Waltham, NJ, USA).

### 2.3. Genetic Analyses

The bi-directional chromatograms were screened using a SeqScanner version 1.0 (Applied Biosystems Inc., Waltham, CA, USA), and the noisy parts were trimmed from both ends to avoid the nuclear mitochondrial DNA segments (NUMTs). The consensus sequences were reviewed through the nucleotide BLAST (https://blast.ncbi.nlm.nih.gov, accessed on 25 November 2022) and ORF finder (https://www.ncbi.nlm.nih.gov/orffinder/, accessed on 25 November 2022) search tools to confirm the appropriate amino acid array of the vertebrate mitochondrial genome. The spatial patterns of genetic divergences were investigated through a genetic landscape shape interpolation analysis using the Alleles in Space v1.0 program [23]. The Kimura-2 parameter (K2P) genetic distances were calculated through the MEGA11 program [24]. The representation of intraspecific genetic distances was plotted through BoxPlot in RStudio (https://posit.co/). To estimate the molecular operational taxonomic units (MOTUs), the Automatic Barcode Gap Discovery (ABGD) and Poisson Tree Processes (PTP) were applied [25,26]. The ABGD analysis was executed on the web server (www.abi.snv.jussieu.fr/public/abgd/, accessed on 25 November 2022) using the Jukes-Cantor (JC69) model. The maximum-likelihood tree was constructed in RAxML v2.0.1 to perform the PTP analysis (http://species.h-its.org/ptp/, accessed on 25 November 2022) [27]. The number of unique haplotypes, the number of polymorphic sites, and haplotype diversity (Hd) were estimated by DnaSP 6 [28]. The TCS networks of all haplotypes were constructed in POP-ART [29,30].

### 2.4. Phylogenetic Inference

A total of 46 mtCytb sequences of *G. elegans* and 7 sequences of *G. platynota* were acquired from GenBank and aligned by ClustalX to build a final dataset (458 bp) [31]. The sequence of *Manouria emys* (DQ080040) was also acquired from GenBank and used as an outgroup in the phylogenetic inference. The suitable model was confirmed using Mr. MODELTEST v2 with the lowest BIC (Bayesian information criterion) score [32]. The Bayesian (BA) phylogeny was built in MrBayes v3.1.2 with a GTR  +  G + I model with one cold and three hot chains and run for 600,000 generations with 25% burn-in, trees saving at every 100 generations, and other default parameters [33]. The generated BA tree was illustrated through the web-based iTOL tool (https://itol.embl.de/) [34].

### 2.5. Species Occurrence Data

**Figure 1:**
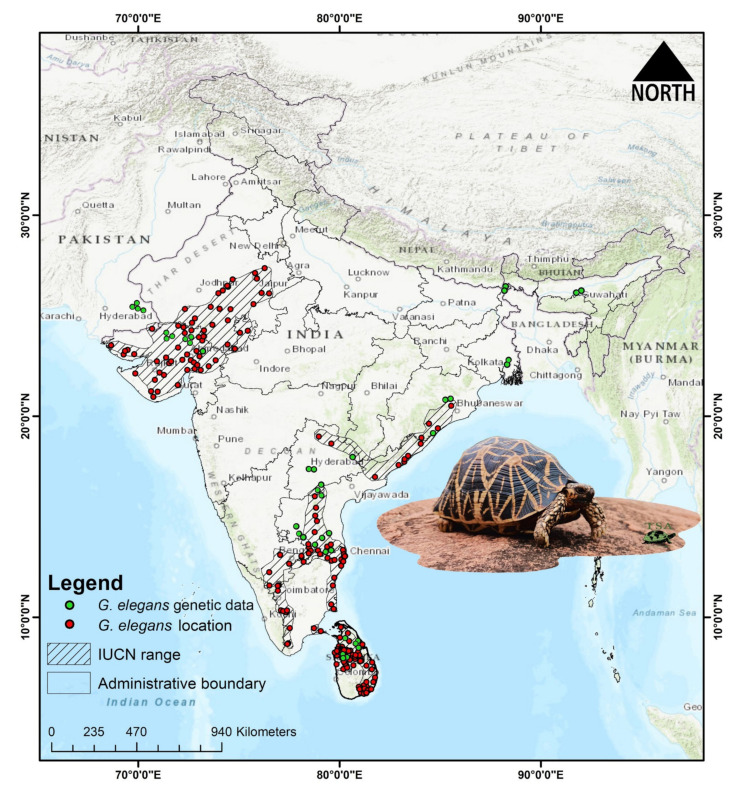
Map showing the distribution of *Geochelone elegans*. The Map is prepared in the ArcGIS 10.6 platform using polygons (shp file) from the IUCN Red List of Threatened Species, which was assessed on 13 March 2018 (IUCN version 2022-1 acquired on 22 November 2022). The known distribution and collection sites of *G. elegans* are marked by red dots. The locations of the *G. elegans* genetic data generated during the current and previous studies are marked by green dots. The species photograph was acquired from the Turtle Survival Alliance India with prior permission.

The spatial occurrence records of *G. elegans* were collected from the TFTSG assessment and associated literature, as well as from the GBIF online data repository (https://doi.org/10.15468/dl.uwhqg8, accessed on 25 November 2022) [2]. We identified (*n* = 205) spatially independent occurrence points for *G. elegans* (Figure 1). We used the SDMtoolbox to remove the spatial autocorrelation, using the locality points with a search radius of 1 km based on the raster resolution of the predictor variable, to reduce the overfitting of the model [35].

### 2.6. Model Covariate Selection

The variables, which may play a significant role in predicting suitable habitats, were selected for primary screening by considering the ecological requirements of *G. elegans* [36]. We started with a set of 24 habitat variables grouped into 4 types: bioclimatic, land cover and land use (LULC), topographic, and anthropogenic (Table S1, Figure S1). The climatic variables were represented by 19 bioclimatic variables from Worldclim v2.0 (https://www.worldclim.org/, accessed on 25 November 2022) [37]. The aridity variable within the study area was acquired from Version 3 of the Global Aridity Index and Potential Evapotranspiration Database [38]. The LULC was acquired from Copernicus Global Land Service (https://lcviewer.vito.be/download, accessed on 25 November 2022). Further, we used the Human Influence Index (HII) to understand human influences on the target species [39]. The topographic variables, i.e., elevation and slope, were generated using the 90 m Shuttle Radar Topography Mission (SRTM) data (http://srtm.csi.cgiar.org/srtmdata/, accessed on 25 November 2022). For the final model run, predictors were resampled at 1 km spatial resolution using spatial analysis within ArcGIS 10.6. We used SDMtoolbox v2.4 to check the spatial multicollinearity among the predictors, and the variables with r > 0.8 Pearson’s correlation were dropped from the final modeling environment (Figure S1).

### 2.7. Model Building and Evaluation

We implemented maximum entropy modeling (MaxEnt) v3.4.4 for the present study, as it is a widely used predictive modeling tool that is known to perform well even if the number of covariates exceeds the number of occurrences for a predictive model [40,41]. We used the bootstrapping replication method and Bernoulli generalized linear model with the ClogLog link function for the present model development [42]. The model used the training data on each occurrence point as n-1 and tested the model performance with the remaining points and 50 runs as replicates [40,43]. The final results generated a probability distribution output as a continuous probability surface raster of the study extent ranging from 0–1, with ‘1’ being the most suitable habitat and ‘0’ being the most unsuitable habitat area for *G. elegans*. The variable influence was estimated using the Jackknife test of developed regularized training gain [44]. For model evaluation, we used the area under the curve (AUC) statistics of the receiver operating characteristic (ROC) curves [45]. The AUC test statistic values ranged from 0 to 1, where a value <0.5 indicated minimum discrimination between the predictive presence and absence areas and was considered to be worse than random, 0.5 indicated a random prediction, 0.7–0.8 indicated an acceptable model, 0.8–0.9 indicated an excellent model, and >0.9 indicated an exceptional model [46,47]. We prepared the binary maps based on a test sensitivity and specificity (SES) threshold equal to the predicted suitable habitat for *G. elegans* to evaluate the zonal statistics and area calculations.

### 2.8. Habitat Quality Assessment

For the habitat quality assessment, we compared the suitable areas of distinct suitable ranges of *G. elegans*. The study used FRAGSTATS v4.2.1 to calculate the class-level landscape metrics using the PLAND (percentage of landscape), the number of patches (NP), patch density (PD), aggregation (AI), largest patch (LPI), total edge (TE), interspersion and juxtaposition (IJI), edge density (ED), and landscape shape (LSI) as the indices of the level of habitat quality and level of fragmentation indicators in the present area [48,49,50].

## 3. Results

### 3.1. Molecular Characterization

**Figure 2:**
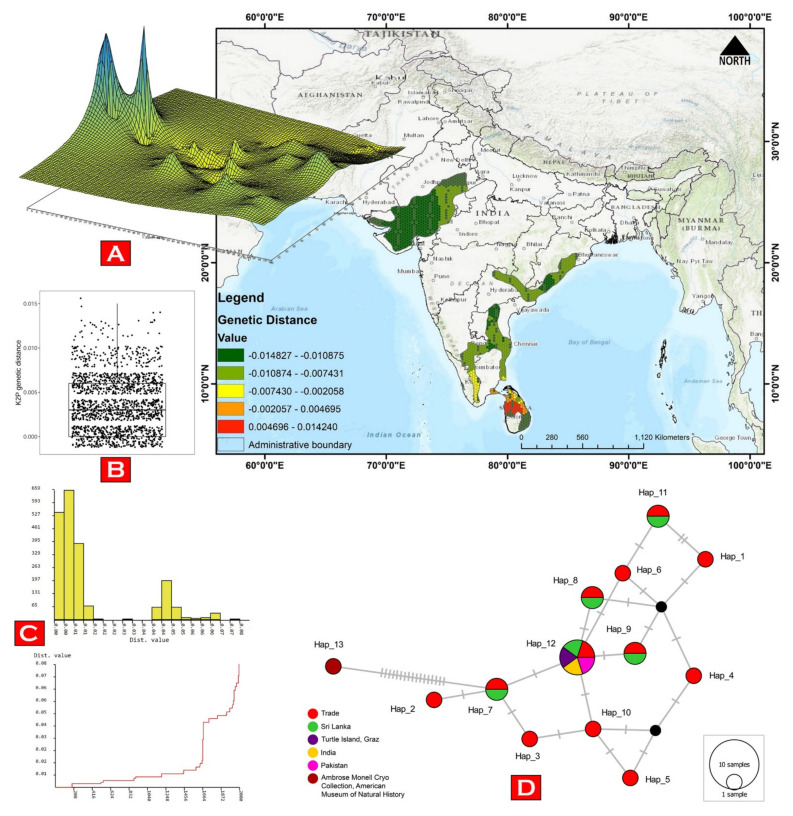
(**A**) Results of the genetic landscape shape interpolation analysis of *G. elegans* with the distance weight parameter α = 1, projected on a grid size of 1 × 1 degree. The high values denote high, and low values denote low, genetic diversity corresponding to their locality information; (**B**) a box plot showing the weak intra-species genetic distance; (**C**) the ABGD web interface of the present dataset showing a histogram and ranked distances; and (**D**) a TCS haplotypic network showing the relationship among all the haplotypes of *G. elegans*. Circle sizes are proportional to the number of individuals that belong to each haplotype, and mutational steps are symbolized by dashes. The median vectors (hypothetical haplotypes) are denoted by black circles.

**Figure 3:**
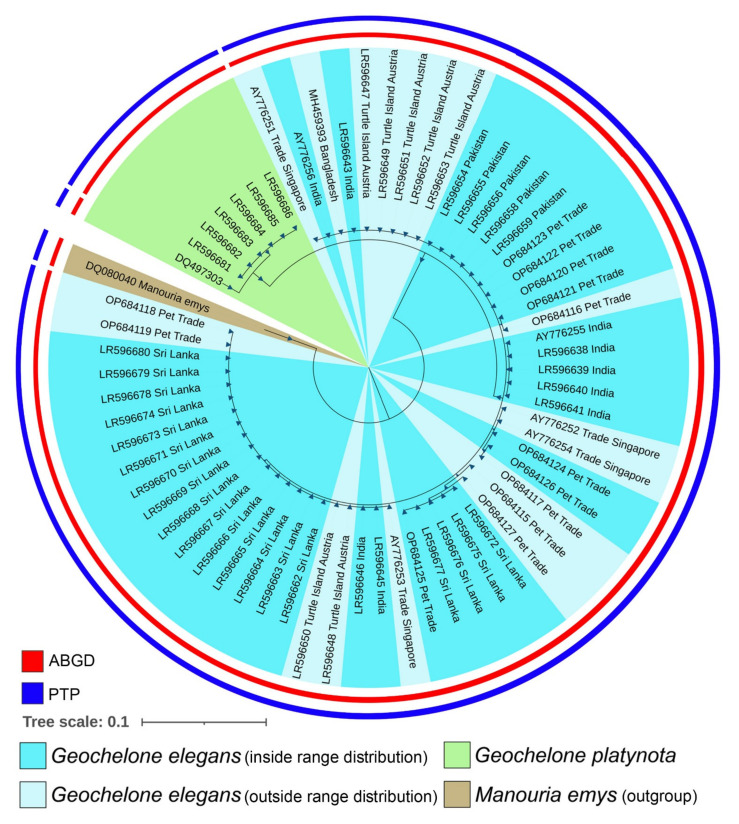
Unified Bayesian (BA) phylogenetic tree, based on the mitochondrial Cytb gene, shows the clustering pattern of *G. elegans* and *G. platynota*. The BA posterior probability support of each node was superimposed and marked by differently sized blue triangles. NCBI accession numbers and collection information are represented, with each node in parentheses. The red- and blue-colored bars indicate delineated MOTUs by ABGD and PTP, respectively.

The generated sequences of *G. elegans* were contributed to the global GenBank database under the accession numbers (OP684115–OP684127). The generated sequences showed 99–100% similarity with the database sequences of the same species. The overall mean K2P genetic distance was 0.6% in the *G. elegans* dataset. The genetic landscape analysis also revealed small zones of low genetic differentiation across the distribution range of *G. elegans* (Table S2, Figure 2A). On a distant geographical scale, the analysis strengthens the earlier hypothesis and enlightens the mixed genetic diversity of *G. elegans* in India and Sri Lanka. The intra-species genetic distance ranged from 0% to 1.5% in the present dataset (Figure 2B). Remarkably, an unexpectedly high intra-species genetic distance (5.2–6.5%) and a high number of segregating sites (*n* = 17) were observed compared with the database sequences (DQ497299) of *G. elegans* vouchered in the Ambrose Monell Cryo Collection, American Museum of Natural History. Hence, this database sequence was not incorporated into multiple species delimitation and phylogenetic analyses. The ABGD and PTP analyses revealed a single MOTU of *G. elegans* in the initial partitioning and maximum-likelihood-supported solutions, respectively (Table S3, Figure 2C). The present dataset of *G. elegans* revealed 13 haplotypes with 22 segregating sites and haplotype diversity (Hd) = 0.7195. The TCS network depicted a mixed haplotypic distribution of *G. elegans* in terms of their collection sites (Figure 2D). The Bayesian (BA) phylogeny showed monophyletic clustering of all generated and database sequences of *G. elegans*. Both *G. elegans* and *G. platynota* showed distinct clustering in the BA phylogeny (Figure 3). Both species delimitation methods revealed similar results with a single MOTU of *G. elegans*, which is concordant with the genetic distance and tree analyses.

### 3.2. Model Performance and Habitat Suitability

**Figure 4:**
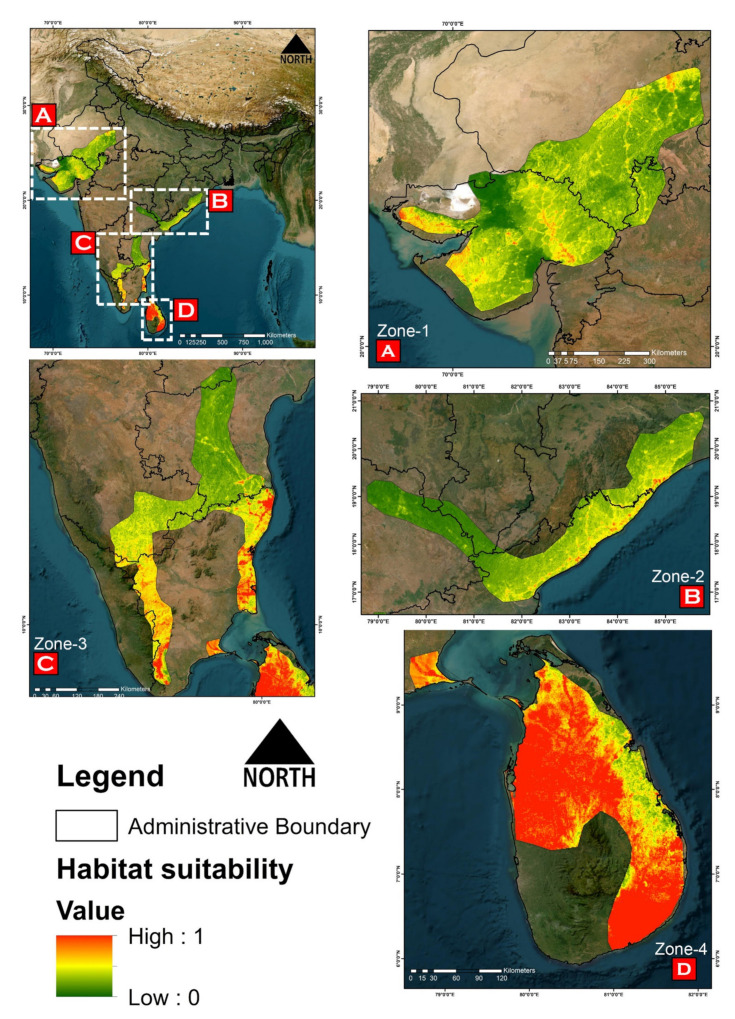
Representing the probability of suitable habitats of *G. elegans* within the distribution range. (**A**) Zone-1 comprising the western range within Gujarat and Rajasthan of India; (**B**) Zone-2 comprising the eastern range within Odisha and Telangana, and the north-eastern portion of Andhra Pradesh of India; (**C**) Zone-3 comprising the southern range within Tamil Nadu and Karnataka, and the central to southern parts of Andhra Pradesh; (**D**) Zone-4 comprising the north-western to south-eastern regions within Sri Lanka.

**Figure 5:**
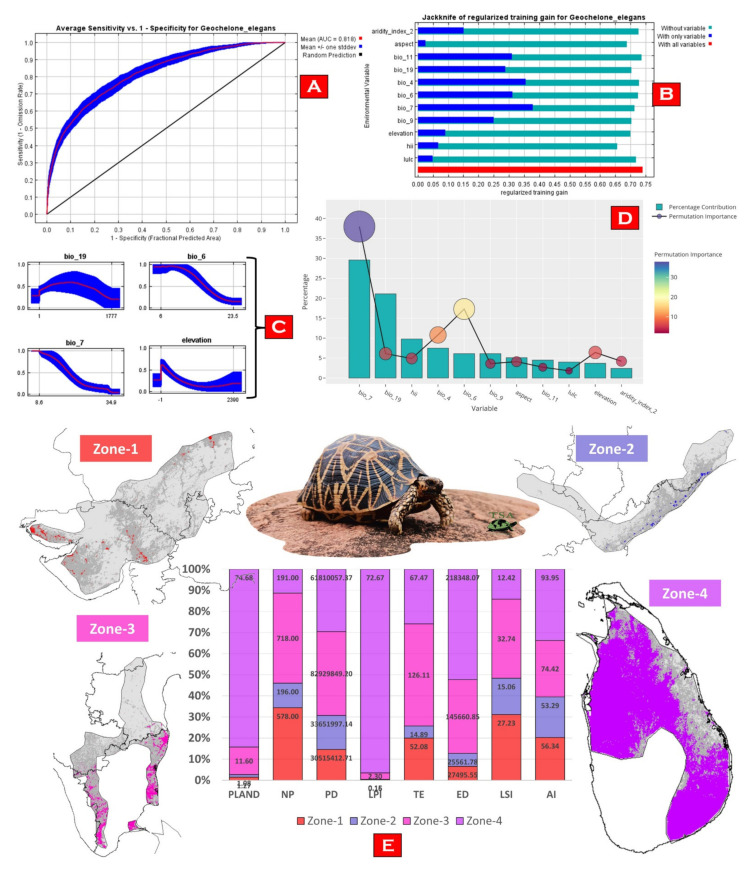
Represents model evaluation, variable influence, and habitat quality assessment of *G. elegans*. (**A**) The average training ROC for the final model replicates. (**B**) Jackknife tests for all ten variables. The Blue bar shows each variable’s importance in explaining the data variation when used separately. The green bar shows the loss in total model gain when the particular variable was dropped, which signifies the unique information necessary for explaining the model. The red bar shows the total model gain. (**C**) Response curves of the important variables for the habitat suitability of *G. elegans*. (**D**) Percentage contribution is represented by a column graph (the color ramp represents the percentage contribution), and permutation importance is represented by the circular plot (size and color ramps represent permutation importance). (**E**) Represents the percentage stack of class-level matrices used for the habitat quality assessment of *G. elegans* in four zones. (PLAND = percentage of landscape; NP = number of patches; PD = patch density; LPI = largest patch index; TE = total edge; ED = edge density; LSI = landscape shape index; IJI = interspersion and juxtaposition index; AI = aggregation index).

The model predicted the suitable habitats for *G. elegans* within the study landscape with excellent accuracy (Figure 4). The average training AUC for replicate runs for the model was found to be 0.818 ± 0.017 (SD) (Figure 5A). Out of the total distribution range extent (570,254 km^2^), about 56,495 km^2^ (9.90%) is suitable for *G. elegans* (Figure 4). The results also suggest the most suitable habitats in Sri Lanka and the southern region of India. The biggest and continuous habitat patch for *G. elegans* was found in the north-western and south-eastern portions of Sri Lanka (36,060 km^2^). Further, in India, the most suitable and continuous habitat patches were distributed in the far southern portion of Andhra Pradesh and Tamil Nadu, with a total area of 15,699 km^2^ (Figure 4). The model suggests that the distribution of habitat patches for *G. elegans* was strongly influenced by temperature annual range, with a relative contribution of 29.6%, followed by the contribution of precipitation of the coldest quarter by 21.1% (Figure 5). Further, human influence was also found to influence the distribution of *G. elegans*, with a percentage contribution of 9.8% (Figure 5).

### 3.3. Habitat Quality Estimation

For the comparative habitat quality estimation between the ranges, we have designated the ranges into a total of four zones, i.e., zone 1 (comprising the western range within Gujarat and Rajasthan in India); zone 2 (comprising the eastern range within Odisha and Telangana, and the north-eastern portion of Andhra Pradesh in India); zone-3 (comprising the southern range of Tamil Nadu and Karnataka, and the central part of Andhra Pradesh in India); and zone 4 (comprising the north-western and south-eastern portions of Sri Lanka) (Figure 5E). Our results suggest that zone 4 constitutes about 74% of the total suitable areas for the species, followed by zone 3 with 11.60%. Habitats within the Sri Lankan range were found to be the best for *G. elegans*, with the lowest score of NP (191) and high values of LPI (72.67), suggesting habitat continuity and less habitat fragmentation (Figure 5E). Moreover, the continuity and habitat quality can be also observed through the comparatively high value of AI (93.95) within the Sri Lankan range. However, zone 3 and zone 1 depicted high NP, PD, TE, and LSI, suggesting a substantial level of habitat fragmentation in the region (Figure 5E).

## 4. Discussion

The Convention on Biological Diversity (CBD) and the Intergovernmental Science-Policy Platform on Biodiversity and Ecosystem Services (IPBES) made us aware of the significant reduction in the rate of biodiversity loss and extinction risks to earth life, driven by anthropogenic activity, invasive alien species, overexploitation, climate change impacts, and ecological collapse [51,52]. To reduce the risks and support the ecosystems, several new unifying concepts and their implementation have been recommended in recent years to achieve worthy conservation actions [53,54]. The global assessments indicate that 25.4% of mammals, 13.6% of birds, 21.1% of reptiles, and 40.7% of amphibians are threatened with extinction [55]. Notably, the conservation priority of reptiles among other tetrapods has always been overlooked, and specific conservation needs have been claimed due to their extraordinary diversity in arid regions [56]. At the same time, some reptiles, such as Testudines, are the most threatened vertebrates, facing high anthropogenic pressure worldwide and requiring urgent and targeted action plans [57]. In many cases, seized turtles and tortoises are moved far from their place of capture and returned to the wild without knowing their true origin. However, finding preferable sites for their translocation is often challenging considering the ecological demands, genetic make-up, competition, and hybridization of confiscated animals. Releasing live animals seized from the illegal wildlife trade into unknown wild populations or close to their distribution range causes the admixture of distinct genetic lineages and increases the potential conservation risks of reintroduction [58,59].

The molecular data have proven to be a successful tool for illuminating various biological questions of Testudines around the globe. The genetic architecture of land tortoises is often used to identify new species [60], detect cryptic variants [61], understand phylogeographic patterns [62,63,64], as well as infer diversity- and evolutionary-based relationships [65,66,67,68]. Complete mitochondrial genome and whole genome data also provide evidence revealing the phylogenetic relationships of these enigmatic species and insights into their longevity [69,70,71]. Because of the historical background of land tortoises, ancient DNA-based analyses also play a special role in determining their evolutionary history [72,73]. The nuclear and mitochondrial gene sequences of *G. elegans* and *G. platynota* were irrespectively generated to examine the evolutionary relationships of Testudines [74,75,76,77,78]. However, a few studies were executed specifically on *G. elegans* and exhibited the loss of genetic diversity among the different populations due to their enormous trade volume [8,9,79,80]. The generated molecular data will be utilized as a reference DNA sequence for examining the confiscated *G. elegans* in near future as well as utilized in wildlife forensics. Nevertheless, the present genetic analysis reveals lost genetic diversity across the distribution range of *G. elegans* with weak intraspecific differences, which is congruent with previous studies. The partial mitochondrial cytochrome b gene sequences were inadequate to distinguish the different populations of *G. elegans* from distant localities. Due to the unscientific release of confiscated animals in the wild, and the subsequent hybridization between different populations over the years, star tortoises have lost genetic diversity and have experienced increases in the vulnerability of wild populations. Beyond the legal restrictions of global biodiversity research, we suggest that the genetic screening of *G. elegans* by other genes be required for Pakistan, India, and Sri Lanka, which would provide a better genetic explanation for their phylogeographic footmark across their distribution range [81,82]. Current research recommends genetic screening to identify founder individuals or isolated adult colonies, in the wild or captive for scientific breeding, to preserve maximum genetic diversity, avoid inbreeding depression, and support the successful reintroduction of captively bred offspring to the wild to recover the lost heterozygosity of *G. elegans*.

**Figure 6:**
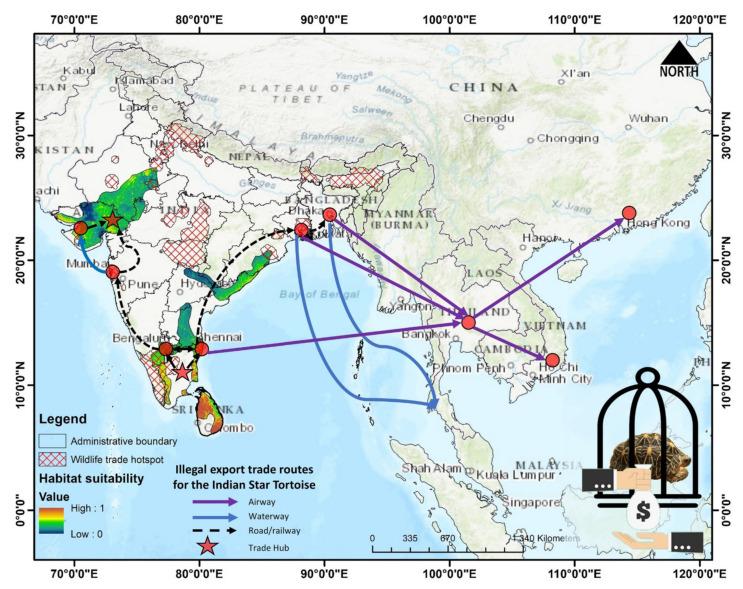
Comparative visualization of the suitable ranges for *G. elegans*, along with illegal wildlife trade hotspots (https://www.wpsi-india.org/crime_maps/trade_hotspots.php, accessed on 25 November 2022) and the domestic and international illegal export trade routes of *G. elegans* (D’Cruze et al., 2015). The species photograph was acquired from the free media repository Wikimedia Commons.

The massive unlawful trade of reptiles, including the Indian star tortoise (*G. elegans*), has reached an alarming level [83,84]. To protect these highly threatened animals in wild, a joint endeavor of the Turtle Survival Alliance (TSA)—a United States-based organization—and associate partners across South Asia is underway to rescue this tortoise species from extinction and involves trade control, captive breeding, head-starting, and a reintroduction to the wild [85]. Our result suggests that about 10% of the area with the IUCN range of the Indian star tortoise is suitable for habitation; however, this area is further subjected to the impacts of human-mediated habitat degradation (Figure 5E). Areas within the states of Gujrat and Rajasthan, followed by Tamil Nadu, Karnataka, and Andhra Pradesh, suffer the most with the highest levels of habitat fragmentation due to the rapid development of urbanization and croplands. The present study elucidates that most of the suitable habitats of *G. elegans* are under significant human pressure, which is an issue that requires special attention for their conservation [86]. Further, to mitigate the existing anthropogenic threats to *G. elegans*, it is also important to have species-specific knowledge about their habits and habitats as well as their trade routes. Previous studies have suggested that areas near Ahmedabad, Bengaluru, and Chennai are illegal trading hubs for Indian star tortoises [3]. While integrating the present SDM results with the findings of D’Cruze et al. (2015), we suspect multiple wildlife trade hubs are overlapped within the suitable range (Figure 6). Hence, we recommend active cooperation between national (state level) and international organizations to prioritize these conflict hotspots. These molecular and habitat-suitability data will be key components in developing improved conservation action plans for the successful reintroduction of captively bred and confiscated star tortoises into the wild. The present study will not only help in understanding the genetic diversity of star tortoises in India and beyond but will also help us understand the genetic impacts of the decimation of this oldest-living animal by humans and provide important guidance for the conservation of the remaining genetic diversity of this threatened species.

## 5. Conclusions

Due to habitat fragmentation, anthropogenic threats, and illegal hunting, star tortoise populations have declined significantly in South Asian countries. Unscientific translocations have led to genetic admixture between different populations and wiped out their phylogeographic differentiation throughout the range. Both mitochondrial Cytb genetic data and MaxEnt species distribution modeling corroborated these facts. We recommend that a comprehensive genetic survey be required to search the isolated wild colonies and subsequent scientific breeding to retrieve their lost genetic diversity. More effective conservation action plans by the Turtle Survival Alliance and other organizations are needed to reduce habitat destruction and precisely identify suitable protected areas for the conservation of this species.

## Data Availability

The nucleotide sequence data that support the findings of this study are openly available in the NCBI GenBank database (https://www.ncbi.nlm.nih.gov) under accession no. OP684115-OP684127.

## Supplementary Materials

The following supporting information can be downloaded at: https://www.mdpi.com/article/10.3390/ani13010150/s1, Figure S1: Representing the final set of variables maps used for the distribution modeling of *G. elegans*; Table S1: Primary environmental and topographical variables used for modeling; Table S2: ABGD and PTP results of the present dataset of *G. elegans* and *G. platynota*; Table S3: Genetic landscape shape interpolation analysis results showing the residual genetic distances used as landscape heights.
