# Supplementary material for: Mitochondrial DNA and Distribution Modelling Evidenced the Lost Genetic Diversity and Wild-Residence of Star Tortoise, Geochelone elegans (Testudines: Testudinidae) in India
Source: Animals (Basel). 2022 Dec 30;13(1):150. doi: 10.3390/ani13010150 (PMC9817980; doi:10.3390/ani13010150)
Supplement: Supplementary file 1 [file animals-13-00150-s001.zip › animals-2097324-supplementary.pdf]

Supplementary File(s)

Mitochondrial DNA and distribution modelling evidenced the lost genetic diversity and wild-residence of Star Tortoise, *Geochelone elegans* (Testudines: Testudinidae) in India

Shantanu Kundu[1†], Tanoy Mukherjee[2†], Ah ran Kim[3], Soo Rin Lee[3], Abhishek Mukherjee[4], Won-Kyo Jung[3], Hyun-Woo Kim[1,3*]

[1]Department of Marine Biology, Pukyong National University, Busan 48513, South Korea.

[2]Agricultural and Ecological Research Unit, Indian Statistical Institute, Kolkata 700108, India.

[3]Research Center for Marine Integrated Bionics Technology, Pukyong National University, Busan 48513, South Korea.

[4]Biological Sciences Division, Indian Statistical Institute, Kolkata 700108, India.

†These authors contributed equally to this work.

* Corresponding author:

Hyun-Woo Kim Ph. D.

Department of Marine Biology, Pukyong National University

48513, Republic of Korea

Tel: 82-51-629-5926, Fax: 82-51-629-5930

E-mail: kimhw@pknu.ac.kr

Table S1. Primary environmental and topographical variables used modelling.

Sl. No.	Code	Variables description
**Bioclimatic**		
1.	bio_1	Annual Mean Temperature
2.	bio_2	Mean Diurnal Range (Mean of monthly (max temp - min temp)
3.	bio_3	Isothermality (BIO2/BIO7) (* 100)
4.	bio_4	Temperature Seasonality (standard deviation *100)
5.	bio_5	Max Temperature of Warmest Month
6.	bio_6	Min Temperature of Coldest Month
7.	bio_7	Temperature Annual Range (BIO5-BIO6)
8.	bio_8	Mean Temperature of Wettest Quarter
9.	bio_9	Mean Temperature of Driest Quarter
10.	bio_10	Mean Temperature of Warmest Quarter
11.	bio_11	Mean Temperature of Coldest Quarter
12.	bio_12	Annual Precipitation
13.	bio_13	Precipitation of Wettest Month
14.	bio_14	Precipitation of Driest Month
15.	bio_15	Precipitation Seasonality (Coefficient of Variation)
16.	bio_16	Precipitation of Wettest Quarter
17.	bio_17	Precipitation of Driest Quarter
18.	bio_18	Precipitation of Warmest Quarter
19.	bio_19	Precipitation of Coldest Quarter
20.	aridity_index_2	Aridity Index
**Topographic**		
21.	elevation	Elevation
22.	aspect	Aspect
**Anthropogenic**		
23.	hii	Human Influence Index
**LULC**		
24.	LULC (Categorical)	Herbaceous Vegetation
		Water Bodies
		Cropland
		Bare land
		Shrubland
		Forest
		Urban

Table S2. ABGD and PTP results of the present dataset of *G. elegans* and *G. platynota*.

<table>
<tr><td colspan="3">ABGD species delimitation results</td></tr>
<tr><td colspan="3">Recursive Partition with prior maximal distance P=1.00e-03) Distance JC69 Jukes-Cantor MinSlope=1.500000</td></tr>
<tr><th>Group</th><th>Number of Sequences</th><th>Details</th></tr>
<tr><td>Group [1]</td><td>58</td><td>OP684115_Pet_Trade
OP684116_Pet_Trade
OP684117_Pet_Trade
OP684118_Pet_Trade
OP684119_Pet_Trade
OP684120_Pet_Trade
OP684121_Pet_Trade
OP684122_Pet_Trade
OP684123_Pet_Trade
OP684124_Pet_Trade
OP684125_Pet_Trade
OP684126_Pet_Trade
OP684127_Pet_Trade
LR596680_Sri_Lanka
LR596679_Sri_Lanka
LR596678_Sri_Lanka
LR596677_Sri_Lanka
LR596676_Sri_Lanka
LR596675_Sri_Lanka
LR596674_Sri_Lanka
LR596673_Sri_Lanka
LR596672_Sri_Lanka
LR596671_Sri_Lanka
LR596670_Sri_Lanka
LR596669_Sri_Lanka
LR596668_Sri_Lanka
LR596667_Sri_Lanka
LR596666_Sri_Lanka
LR596665_Sri_Lanka
LR596664_Sri_Lanka
LR596663_Sri_Lanka
LR596662_Sri_Lanka
LR596659_Pakistan
LR596658_Pakistan
LR596656_Pakistan
LR596655_Pakistan
LR596654_Pakistan
LR596653_Turtle_Island_Austria
LR596652_Turtle_Island_Austria
LR596651_Turtle_Island_Austria
LR596650_Turtle_Island_Austria
LR596649_Turtle_Island_Austria
LR596648_Turtle_Island_Austria
LR596647_Turtle_Island_Austria
LR596646_India</td></tr>
</table>

		LR596645_India<br>LR596643_India<br>LR596641_India<br>LR596640_India<br>LR596639_India<br>LR596638_India<br>MH459393_Bangladesh<br>AY776256_India<br>AY776255_India<br>AY776254_Trade_Singapore<br>AY776253_Trade_Singapore<br>AY776252_Trade_Singapore<br>AY776251_Trade_Singapore
Group [2]	1	LR596686_Geochelone_platynota
Group [3]	1	DQ497303_Geochelone_platynota
Group [4]	5	LR596685_Geochelone_platynota<br>LR596684_Geochelone_platynota<br>LR596683_Geochelone_platynota<br>LR596682_Geochelone_platynota<br>LR596681_Geochelone_platynota
Initial Partition with prior maximal distance P=1.00e-03 ; Barcode gap distance = 0.024<br>Distance JC69 Jukes-Cantor MinSlope=1.500000		
Group [1]	58	OP684115_Pet_Trade<br>OP684116_Pet_Trade<br>OP684117_Pet_Trade<br>OP684118_Pet_Trade<br>OP684119_Pet_Trade<br>OP684120_Pet_Trade<br>OP684121_Pet_Trade<br>OP684122_Pet_Trade<br>OP684123_Pet_Trade<br>OP684124_Pet_Trade<br>OP684125_Pet_Trade<br>OP684126_Pet_Trade<br>OP684127_Pet_Trade<br>LR596680_Sri_Lanka<br>LR596679_Sri_Lanka<br>LR596678_Sri_Lanka<br>LR596677_Sri_Lanka<br>LR596676_Sri_Lanka<br>LR596675_Sri_Lanka<br>LR596674_Sri_Lanka<br>LR596673_Sri_Lanka<br>LR596672_Sri_Lanka<br>LR596671_Sri_Lanka<br>LR596670_Sri_Lanka<br>LR596669_Sri_Lanka<br>LR596668_Sri_Lanka<br>LR596667_Sri_Lanka<br>LR596666_Sri_Lanka<br>LR596665_Sri_Lanka

		LR596664_Sri_Lanka<br>LR596663_Sri_Lanka<br>LR596662_Sri_Lanka LR596659_Pakistan<br>LR596658_Pakistan LR596656_Pakistan<br>LR596655_Pakistan LR596654_Pakistan<br>LR596653_Turtle_Island_Austria<br>LR596652_Turtle_Island_Austria<br>LR596651_Turtle_Island_Austria<br>LR596650_Turtle_Island_Austria<br>LR596649_Turtle_Island_Austria<br>LR596648_Turtle_Island_Austria<br>LR596647_Turtle_Island_Austria<br>LR596646_India<br>LR596645_India<br>LR596643_India<br>LR596641_India<br>LR596640_India<br>LR596639_India<br>LR596638_India MH459393_Bangladesh<br>AY776256_India<br>AY776255_India<br>AY776254_Trade_Singapore<br>AY776253_Trade_Singapore<br>AY776252_Trade_Singapore<br>AY776251_Trade_Singapore
Group [2]	6	LR596686_Geochelone_platynota<br>LR596685_Geochelone_platynota<br>LR596684_Geochelone_platynota<br>LR596683_Geochelone_platynota<br>LR596682_Geochelone_platynota<br>LR596681_Geochelone_platynota
Group [3]	1	DQ497303_Geochelone_platynota
**PTP species delimitation results:**<br>Maximum likelihood solution # Max likilhood partition		
**Species**	**Support**	**Details**
Species 1	1.000	DQ080040_Manouria_emys
Species 2	1.000	DQ497303_Geochelone_platynota
Species 3	0.183	LR596685_Geochelone_platynota,<br>LR596684_Geochelone_platynota,<br>LR596683_Geochelone_platynota,<br>LR596682_Geochelone_platynota,<br>LR596686_Geochelone_platynota,<br>LR596681_Geochelone_platynota
Species 4	0.993	LR596640_India,<br>LR596639_India,<br>LR596641_India,<br>LR596643_India,<br>LR596645_India,<br>LR596646_India,<br>LR596648_Turtle_Island_Austria,<br>LR596649_Turtle_Island_Austria,

		LR596650_Turtle_Island_Austria,<br>LR596651_Turtle_Island_Austria,<br>LR596652_Turtle_Island_Austria,<br>LR596653_Turtle_Island_Austria,<br>LR596654_Pakistan,<br>LR596655_Pakistan,<br>LR596656_Pakistan,<br>LR596658_Pakistan,<br>LR596659_Pakistan,<br>LR596663_Sri_Lanka,<br>LR596664_Sri_Lanka,<br>LR596665_Sri_Lanka,<br>LR596666_Sri_Lanka,<br>LR596671_Sri_Lanka,<br>LR596673_Sri_Lanka,<br>LR596679_Sri_Lanka,<br>LR596638_India,<br>MH459393_Bangladesh,<br>AY776256_India,<br>AY776255_India,<br>AY776253_Trade_Singapore,<br>AY776251_Trade_Singapore,<br>AY776254_Trade_Singapore,<br>AY776252_Trade_Singapore,<br>OP684124_Pet_Trade,<br>OP684119_Pet_Trade,<br>OP684116_Pet_Trade,<br>OP684117_Pet_Trade,<br>OP684126_Pet_Trade,<br>OP684121_Pet_Trade,<br>LR596662_Sri_Lanka,<br>OP684115_Pet_Trade,<br>OP684127_Pet_Trade,<br>OP684118_Pet_Trade,<br>OP684122_Pet_Trade,<br>LR596647_Turtle_Island_Austria,<br>OP684123_Pet_Trade,<br>LR596680_Sri_Lanka,<br>LR596678_Sri_Lanka,<br>LR596674_Sri_Lanka,<br>LR596670_Sri_Lanka,<br>LR596669_Sri_Lanka,<br>LR596668_Sri_Lanka,<br>LR596667_Sri_Lanka,<br>OP684120_Pet_Trade,<br>OP684125_Pet_Trade,<br>LR596677_Sri_Lanka,<br>LR596676_Sri_Lanka,<br>LR596675_Sri_Lanka,<br>LR596672_Sri_Lanka

Highest Bayesian supported solution # Most supported partition found by simple heuristic search		
Species 1	1.000	DQ080040_Manouria_emys
Species 2	1.000	DQ497303_Geochelone_platynota
Species 3	0.993	LR596640_India,<br>LR596639_India,<br>LR596641_India,<br>LR596643_India,<br>LR596645_India,<br>LR596646_India,<br>LR596648_Turtle_Island_Austria,<br>LR596649_Turtle_Island_Austria,<br>LR596650_Turtle_Island_Austria,<br>LR596651_Turtle_Island_Austria,<br>LR596652_Turtle_Island_Austria,<br>LR596653_Turtle_Island_Austria,<br>LR596654_Pakistan,<br>LR596655_Pakistan,<br>LR596656_Pakistan,<br>LR596658_Pakistan,<br>LR596659_Pakistan,<br>LR596663_Sri_Lanka,<br>LR596664_Sri_Lanka,<br>LR596665_Sri_Lanka,<br>LR596666_Sri_Lanka,<br>LR596671_Sri_Lanka,<br>LR596673_Sri_Lanka,<br>LR596679_Sri_Lanka,<br>LR596638_India,<br>MH459393_Bangladesh,<br>AY776256_India,<br>AY776255_India,<br>AY776253_Trade_Singapore,<br>AY776251_Trade_Singapore,<br>AY776254_Trade_Singapore,<br>AY776252_Trade_Singapore,<br>OP684124_Pet_Trade,<br>OP684119_Pet_Trade,<br>OP684116_Pet_Trade,<br>OP684117_Pet_Trade,<br>OP684126_Pet_Trade,<br>OP684121_Pet_Trade,<br>LR596662_Sri_Lanka,<br>OP684115_Pet_Trade,<br>OP684127_Pet_Trade,<br>OP684118_Pet_Trade,<br>OP684122_Pet_Trade,<br>LR596647_Turtle_Island_Austria,<br>OP684123_Pet_Trade,<br>LR596680_Sri_Lanka,<br>LR596678_Sri_Lanka,<br>LR596674_Sri_Lanka,

		LR596670_Sri_Lanka, LR596669_Sri_Lanka, LR596668_Sri_Lanka, LR596667_Sri_Lanka, OP684120_Pet_Trade, OP684125_Pet_Trade, LR596677_Sri_Lanka, LR596676_Sri_Lanka, LR596675_Sri_Lanka, LR596672_Sri_Lanka
Species 4	0.817	LR596685_Geochelone_platynota
Species 5	0.626	LR596684_Geochelone_platynota
Species 6	0.436	LR596683_Geochelone_platynota
Species 7	0.246	LR596682_Geochelone_platynota
Species 8	0.190	LR596686_Geochelone_platynota, LR596681_Geochelone_platynota

Table S3. Genetic landscape shape interpolation analysis results showing the residual genetic distances used as landscape heights.

X-coord	Y-coord	Height
72.365	23.48	-1.09170982625668E-02
74.69	18.315	-1.36517334256465E-02
75.485	18.115	-9.98555883019389E-03
89.97	26.29	-7.4014159812967E-03
88.31	24.51	-5.10758410843002E-03
90.03	24.38	-5.52442013713364E-03
70.68	24.39	-1.51862029460058E-02
73.8	19.025	-1.38844755249351E-02
78.54	15.375	-1.00637621187474E-02
78.225	15.045	-1.32278160088791E-02
78.125	13.28	-1.04641633109542E-02
87.065	21.45	-5.7258493175152E-03
85.765	20.065	-1.05296355197483E-02
87.07	21.215	-3.49147293775031E-03
79.015	14.245	-8.16085422948892E-03
79.045	13.725	-1.04190412482271E-02
79.62	14.36	-1.15344721287776E-02
87.005	23.36	-7.42482694417526E-03
75.9	20.21	-8.29243980585676E-03
79.03	13.45	-9.87415115871616E-03
79.635	13.565	-1.10632247192882E-02
80.37	8.065	1.41240616232136E-02
79.165	10.7	-1.38093240313337E-02
79.015	10.315	1.56046887820829E-02
75.89	20.34	-7.23304451872744E-03
78.63	17.27	-6.19464889220315E-03
80.685	8.245	1.48266336274729E-02

80.835	8.63	-1.26347135545802E-02
79.94	15.85	-1.35640786318919E-02
79.97	15.33	-1.34136545159453E-02
80.07	10.87	-1.18547538512319E-02
78.715	13.12	-1.30574853127513E-02
83.46	14.32	-7.94462022349874E-03
84.76	15.705	-4.37381081891991E-03
79.765	16.4	-1.58602568972185E-02
79.465	16.98	-1.36314632461586E-02
78.94	16.56	-0.013679407760595
80.385	11.05	-9.8223099579217E-03
83.03	18.325	-1.29628440076606E-02
82.71	16.835	-1.04197876842861E-02
82.695	16.56	-9.80916019788871E-03
78.84	14.795	-1.02221745539683E-02
79.415	15.43	-1.37013185928426E-02
70.665	24.455	-0.012109046747533
72.35	23.545	-1.36365290669613E-02
71.475	24.19	-1.11498496796496E-02
82.19	18.85	-6.20858286870256E-03
79.455	17.11	-8.15957137481711E-03
83.025	18.56	-1.18354775588833E-02
71.555	23.745	-1.18545437902989E-02
79.02	25.76	-6.97669913162564E-03
80.705	24.85	-3.00884207403978E-03
79.46	21.79	-6.58343363318368E-03

Figure S1. Representing the final set of variables maps used for the distribution modelling of *G. elegans*.

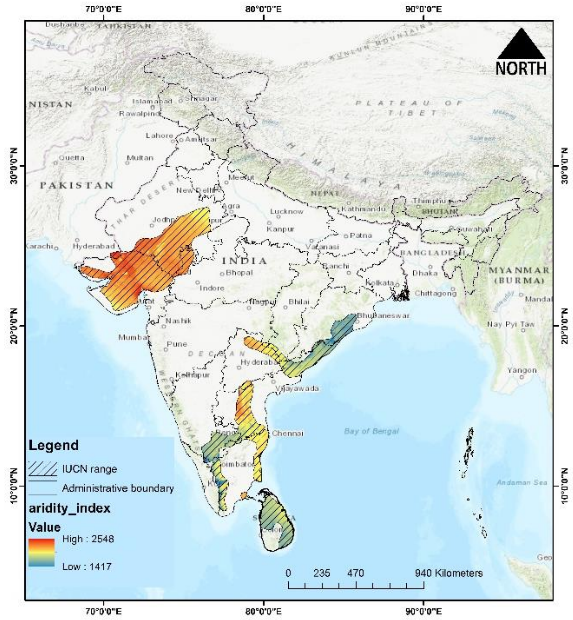

Aridity Index

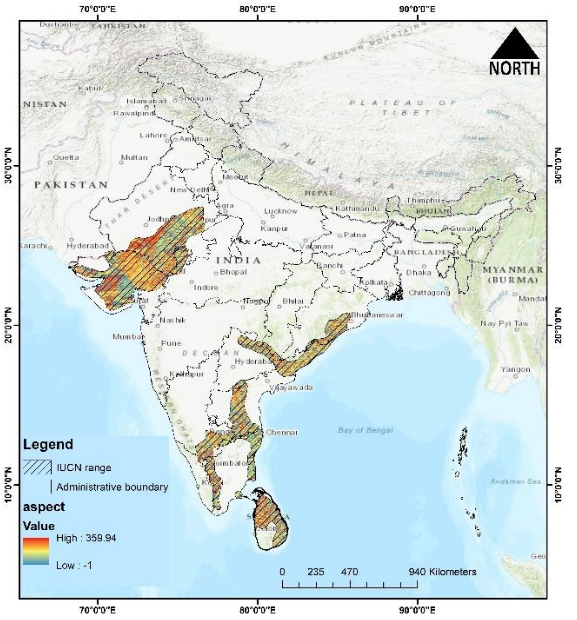

Aspect

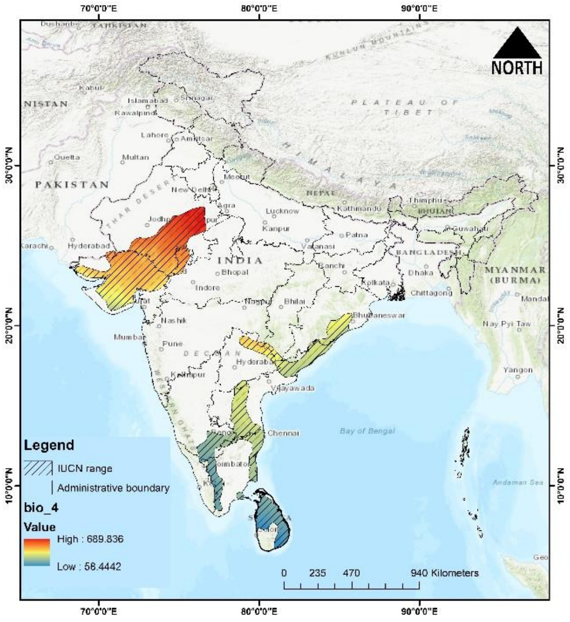

Bio_4 [Temperature Seasonality (Standard Deviation)]

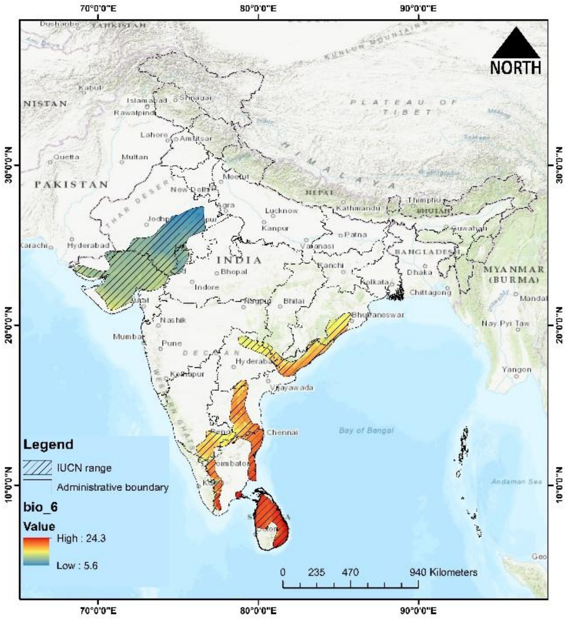

Bio_6 [Min Temperature of Coldest Month]

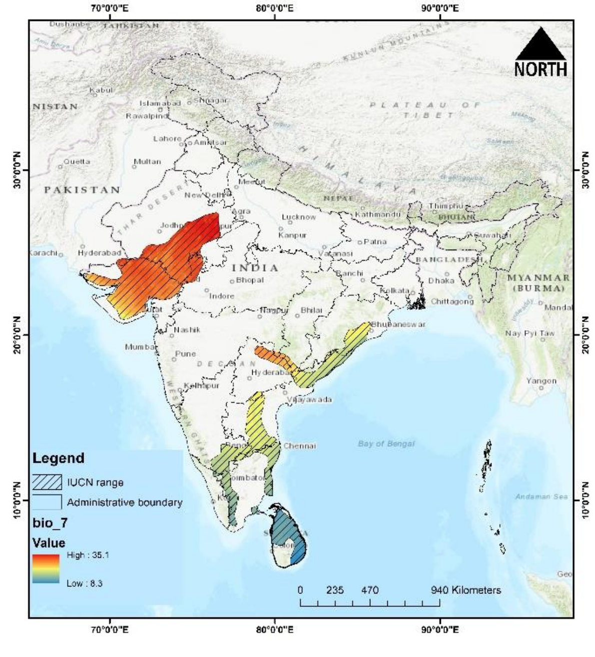

Bio_7 [Temperature Annual Range (BIO5-BIO6)]

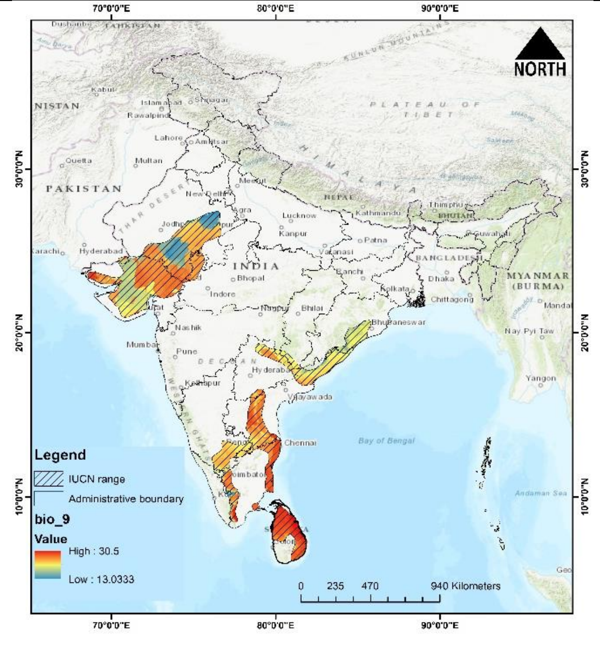

BIO_9 [Mean Temperature of Driest Quarter]

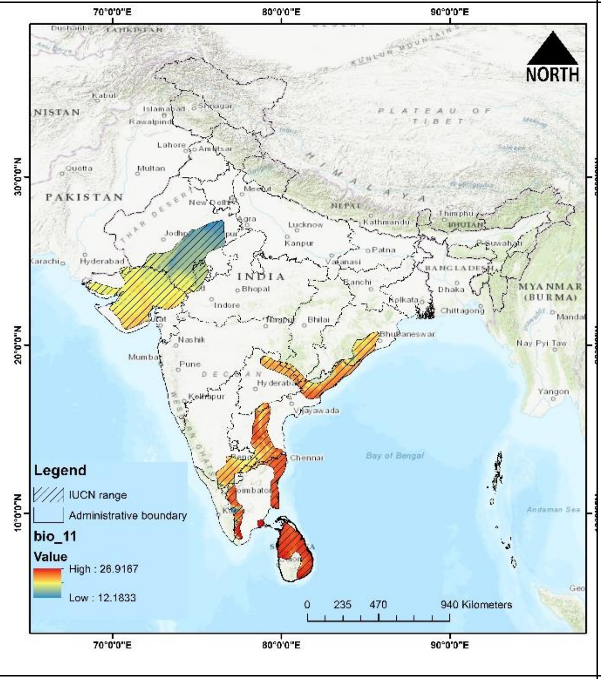

BIO_11 [Mean Temperature of Coldest Quarter]

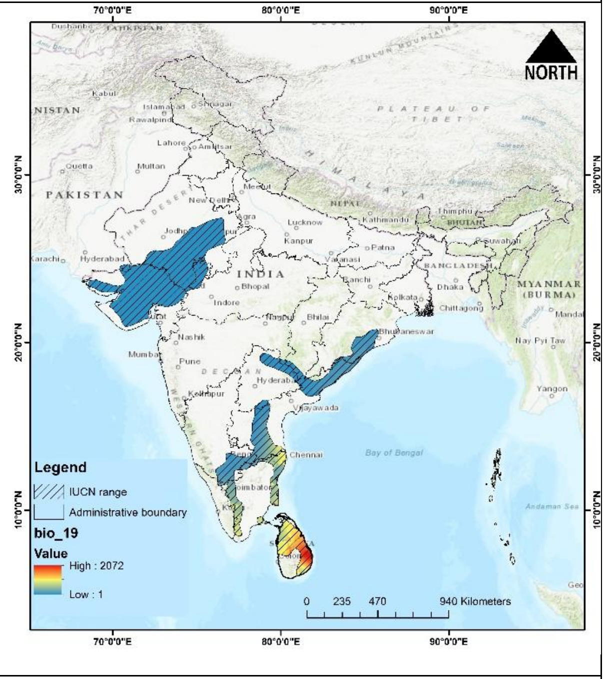

BIO_19 [Precipitation of Coldest Quarter]

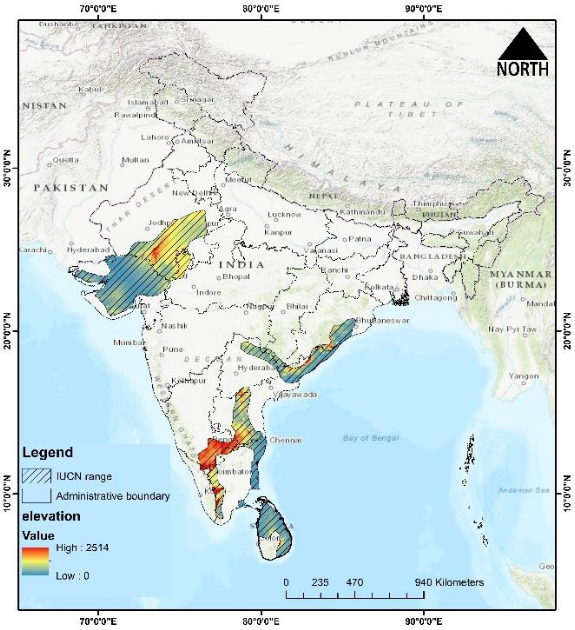

Elevation

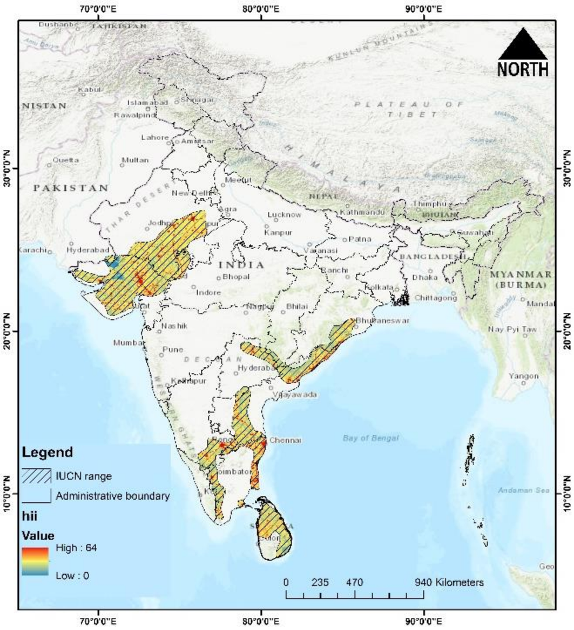

Human Influence Index [HII]

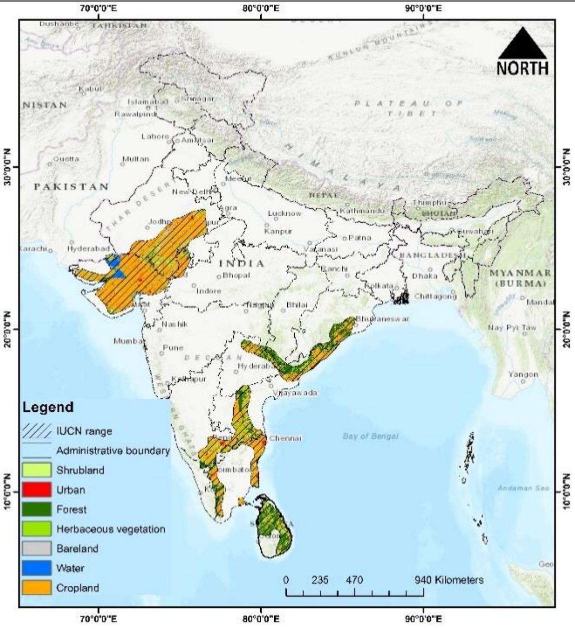

LULC [Landuse Landcover]
